# Meta-analysis of postoperative adjuvant therapy for small bowel adenocarcinoma

**DOI:** 10.1371/journal.pone.0200204

**Published:** 2018-08-10

**Authors:** Xiaojian Ye, Guoqiang Zhang, Haibin Chen, Yong Li

**Affiliations:** 1 Department of Surgery, Shangrao First People's Hospital, Shangrao Jiangxi Province, China; 2 Department of General Surgery, The First Affiliated Hospital of Nanchang University, Yongwai Zhengjie, Nanchang, Jiangxi Province, China; Universita degli Studi di Napoli Federico II, ITALY

## Abstract

**Objective:**

The role of adjuvant therapy in small bowel adenocarcinoma (SBA), a rare malignancy with a poor prognosis, is controversial. The purpose of this article is to investigate the impact of adjuvant therapy on the survival of patients with SBA in a meta-analysis.

**Methods:**

We performed a comprehensive search of PubMed, EMBASE and the Cochrane Library database between 2010 and 2017. Hazard ratios (HR) with 95% confidence intervals (95%CI) were used to assess the effect of adjuvant chemotherapy and/or radiation treatment after curative surgery in patients with SBA. Moreover, impact of age, sex, stage, differentiation, lymph node involvement, and margin status was also evaluated.

**Results:**

We included 15 studies to evaluate the effect of adjuvant therapy on the survival of patients with SBA. The pooled HR of overall survival (OS) involving 5986 patients showed that adjuvant therapy did not have a statistically significant effect on the survival of patients with SBA (pooled HR = 0.89, 95% CI = 0.73–1.09, p = 0.25). Further, 607 patients with duodenal adenocarcinoma (DA) had similar results (pooled HR = 0.96, 95% CI = 0.75–1.23, p = 0.77). Similarly, adjuvant treatment vs. non-adjuvant treatment in terms of disease-free survival (DFS) or relapse-free survival (RFS) showed the same results (pooled HR = 0.89, 95% CI = 0.64–1.23, p = 0.48). However, we found that adjuvant therapy resulted in favorable postoperative survival in Europe according to the subgroup analysis (pooled HR = 0.63, 95% CI = 0.5–0.8, p = 0.0002). In addition, the pooled HR shows that stage, differentiation, lymph node involvement, and margin status were related to the OS of patients with SBA.

**Conclusion:**

Patients with SBA who received adjuvant therapy after surgery did not receive a significant survival benefit. Adjuvant therapy may be more useful in advanced cancer or metastatic patients.

## Introduction

Small bowel adenocarcinoma (SBA) is a malignant tumor of the small intestine mucosa that is mostly located around the duodenal papilla. SBA as a rare cancer is the most common intestinal malignancy and accounts for about 40% of small bowel tumors. The most SBA arise in the duodenum, which is duodenal adenocarcinoma [1, 2]. In recent years, the global incidence of intestinal adenocarcinoma has increased [3, 4]. At present, the diagnosis and treatment of SBA requires improvement, which has also led to a poor prognosis in small bowel cancer; the 5-year survival rate is about 30% [5, 6]. Therefore, it is urgently needed for general surgeons to explore new treatment modalities in patients with SBA.

In recent years, a large number of adjuvant treatments, including chemotherapy and radiotherapy, have been used in a variety of cancers. In addition, the favorable effect of adjuvant treatments on long-term survival and recurrence has been well acknowledged in non-small cell lung cancer, gastric cancer, breast cancer, colon cancer, and ampulla of Vater cancer [7–11]. Currently, surgical resection is still the main treatment for the patients with SBA. However, more and more adjuvant therapies are being used in the treatment of patients with SBA because of its poor prognosis and high risk of relapse [12, 13]. Chemotherapy is the main therapeutic strategy in patients with SBA, colon adenocarcinoma, or upper gastrointestinal tumors. Globally, the combinations of 5-fluorouracil (5-FU) with either cisplatin, oxaliplatin, or irinotecan are frequently used in the more common gastrointestinal tract tumors; these combinations have been tried in SBA with varying degrees of success [14, 15]. In the meantime, more and more studies were performed to explore the impact of adjuvant therapy on the survival of patients with SBA. However, these studies have not shown consistent results regarding the impact of adjuvant therapy. The effect of adjuvant therapy on the survival of patients with SBA is still not completely clear. Thus, we need to investigate the effect of AT on the survival of patients with SBA in a meta-analysis.

In this meta-analysis, we explore the effect of adjuvant chemotherapy on the OS and DFS/RFS of SBA patients. In addition, we evaluate the impact of other related metrics on the OS of SBA patients.

## Materials and methods

### Search strategy

The PubMed, Embase, and Cochrane Library database articles published in English were searched for eligible studies from January 1, 2010 to December 5, 2017(No significant changes in SBA treatment since 2010). The search was performed with the following terms and their combinations “operation or surgical or surgery,” “adjuvant or chemotherapy or chemoradiation or radiotherapy,”“overall survival or progression-free survival or disease-free survival or relapse-free survival or recurrence-free survival,” “small bowel adenocarcinoma or small bowel tumor or duodenal adenocarcinoma.” References of the acquired articles were manually searched for additional studies.

### Study selection

Related articles were independently reviewed by two authors and selected studies met all of the following inclusion criteria: 1) must be published in English; 2) all included patients had SBA or DA; 3) studies must explore the effect of AC on the OS and DFS/RFS of SBA patients; 4) patients receiving postoperative adjuvant therapy served as the experimental group and patients only undergoing surgery were the control group; 5) log-hazard ratios (HR) and 95% confidence intervals (CI) could be extracted directly or indirectly from articles. The exclusion criteria were as follows: 1) inappropriate article types, including review articles, letters, case reports, editorials, and conference abstracts; 2) tumor research on non-small bowel adenocarcinoma; 3)no set experimental group and control group that met the standards; 4) unable to obtain relevant HR and CI from the data in the article.

### Data extraction and quality assessment

Data including the name of the first author, publication year, country, tumor type, number of patients, tumor stage, tumor differentiation, margin status, statistical method, lymph node involvement, and adjuvant treatment details and the related HR and 95% CI were extracted by two independent reviewers. The study quality was assessed independently according to the Newcastle–Ottawa Quality assessment scale (case-control studies). A higher score indicates a higher quality and the maximum score is 9 points.

### Statistical analysis

We used HRs and their 95% CIs as the effect size to analyze the impact of adjuvant therapy on the survival of patients with SBA. There are two ways to obtain HRs and their 95% CIs; one is obtained directly by the article. The other is to use available data, number of events, and the log-rank statistic to calculate the effect size as described by Tierney et al., or to obtain data from survival curves (data are extracted using Engauge Digitizer software) [16]. We believe that the effect size of direct extraction is more accurate than that indirectly obtained. The I^**2**^ statistic and Chi-squared tests are used to evaluate statistical heterogeneity between the included studies. Substantial heterogeneity was found when I^**2**^ >50% or p<0.05. The statistical model selection was based on whether or not there was heterogeneity. A random effects model was used to eliminate the effects of heterogeneity and a fixed effect model was applied when heterogeneity does not exist. Subgroup analysis was further performed to explore the sources of heterogeneity. Subgroup analyses were carried out according to the following categories: country, scale of study, adjuvant therapy methods, and statistical methods. In addition, sensitivity analysis was conducted to evaluate the stability of the results by the successive omission of individual studies. Results were considered statistically significant when the corresponding 95% CI did not by more than 1 and the P values were less than 0.05. Publication bias was assessed using funnel plots with Egger’s test and Begg’s test. We determined that no publication bias existed when the test P value was>0.05 or when there was funnel diagram symmetry. We used Review Manager (RevMan) Version 5.3 and Stata12.0 software for all statistical analyses in this meta-analysis.

## Results

### Included studies

**Fig 1:**
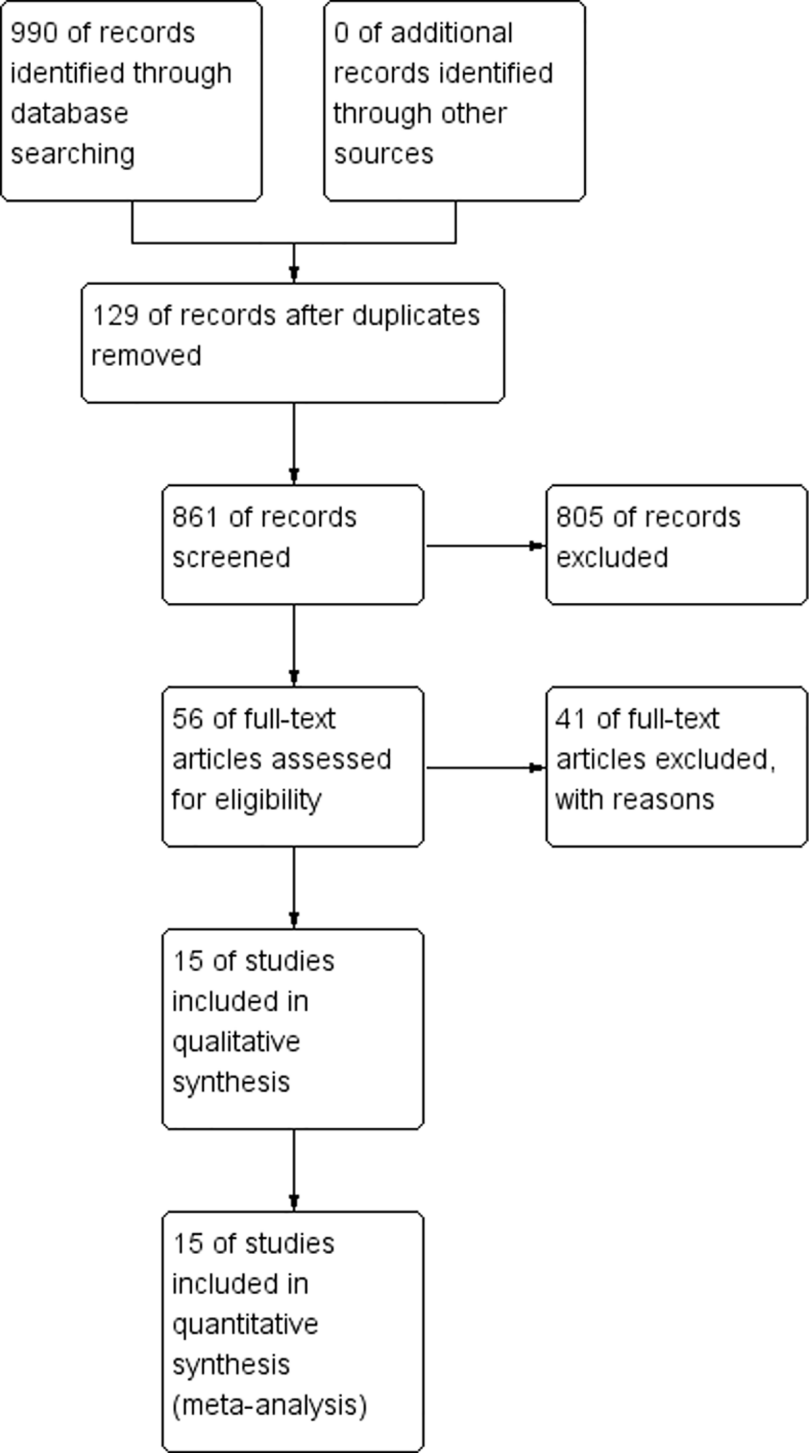
Meta-analysis flow diagram.

A total of 990 relevant studies were identified, including 129 duplicates, using the first search in PubMed, Embase, and Cochrane Library database. Eight hundred and five studies were additionally excluded after screening the titles and abstracts. Fifteen studies [17–31] were retained after reading the full text. Two studies [31, 22] described the same data analysis, so we only included the later study [31] in the subgroup analysis of DA. The article retrieval flow diagram is shown in Fig 1.

### Characteristics of included studies

Table 1 shows the main characteristics of the selected 15 eligible studies. In this table, a total of 15 studies involved 6242 patients who come from China, the United States, South Korea, UK, Turkey, Austria, the Netherlands, and France. Ten articles involving 5635 patients with SBA and 5 articles involving 5635 patients with SBA and 607 patients with DA were included in this analysis. The data of 4 studies were from the national database and the rest came from institutional data. There were 11 studies of adjuvant chemotherapy and 4 studies of adjuvant chemoradiation. Eight studies only provided the effect of adjuvant therapy on the OS of patients and 7 studies used OS and RFS/DFS as the indicator to assess the effect of adjuvant therapy. In statistical methods, HRs and their 95% CIs of 5 studies came from multivariate (MU) analysis and the rest used univariate (UV) analysis in their calculations. We obtained the HR and 95% CI directly from the article in 11 studies and there were only 4 studies from which we needed to calculate the HR and 95% CI indirectly using the available data or survival curves; this adds credibility to our analysis. All of the selected eligible studies scored above 6 points in the quality assessment, which means that the quality of the articles is high.

**Table 1:** Characteristics of included studies.

Reference	Study period	Country	Tumor type	Data Sources	Sample size	Stage	Method	Analysis	Outcome	data	NOS
Wu et al[17]	1999–2008	China	DA	Institutional	141	I-IV	CR	UV	OS	indirect	9
Schwameis et al[18]	1994–2012	Austria	SBA	Institutional	26	NR	CR	UV	OS	indirect	6
Guo et al[19]	2000–2011	China	SBA	Institutional	149	NR	CR	UV	OS	indirect	8
Young et al[20]	1992–2010	United States	SBA	National	1644	I-IV	CR	MU	OS	direct	9
Kanhan et al[21]	1996–2011	Britain	SBA	Institutional	48	ES	CR	UV	OS/RFS	direct	8
Ecker et al[22]	1998–2011	United States	SBA	National	2297	I-IV	CR	UV	OS	direct	8
Legue et al[23]	1999–2013	Netherlands	SBA	National	1194	I-IV	CR	MU	OS	direct	8
Fu et al[24]	1997–2009	United States	DA	Institutional	64	III	CRT	MU	OS/DFS	direct	7
Aydin et al[25]	2003–2013	Turkey	SBA	Institutional	78	I-IV	CR	UV	OS/DFS	direct	9
Kim et al[26]	1991–2002	Korea	DA	Institutional	24	I-IV	CRT	UV	OS/RFS	indirect	8
Koo et al[27]	1989–2009	Korea	SBA	Institutional	52	I-IV	CR	MU	OS/DFS	direct	7
Overman et al[28]	1990–2006	United States	SBA	Institutional	54	I-IV	CRT	MU	OS/DFS	direct	8
Poultsides et al[29]	1984–2006	United States	DA	Institutional	122	I-IV	CRT	MU	OS	direct	9
Zaanan et al[30]	1996–2008	France	SBA	Institutional	93	NR	CR	UV	OS/RFS	direct	9
Ecker et al[31]	1998–2011	United States	DA	National	256	NR	CR	UV	OS	direct	9

SBA: small bowel adenocarcinoma; DA: duodenal adenocarcinoma; NR: not reported; ES: Early stage; CR: Chemotherapy; CRT: Chemoradiation; UV: univariate; MU: multivariate; OS: overall survival; RFS: recurrence-free survival; DFS: disease-free survival; NOS: Newcastle-Ottawa Scale. NOS: Newcastle-Ottawa Scale.

### Main results

**Fig 2:**
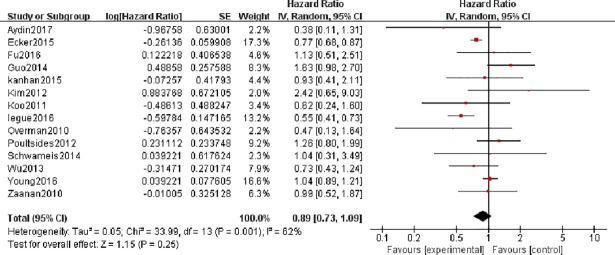
Forest plots to assess the effect of adjuvant therapy on the overall survival of patients with small bowel adenocarcinoma.

The forest plot of 14 studies involving 5986 patients shows that adjuvant therapy did not have a statistically significant effect on the OS of patients with SBA (pooled HR = 0.89, 95% CI = 0.73–1.09, p = 0.25) (Fig 2). Because of the heterogeneity, we used a random effect model to pool effect size (I^**2**^ **=** 62% and P = 0.001). We also conducted subgroup analyses and sensitivity analysis to explore the sources of heterogeneity.

In addition, 5 studies involving 607 patients with HRs and 95% CIs of OS were selected for aggregated survival analysis, which showed similar results (pooled HR = 0.96, 95% CI = 0.75–1.23, p = 0.77) in patients with DA. We did not find heterogeneity in this analysis (I^**2**^ = 22% and P = 0.28).

**Fig 3:**
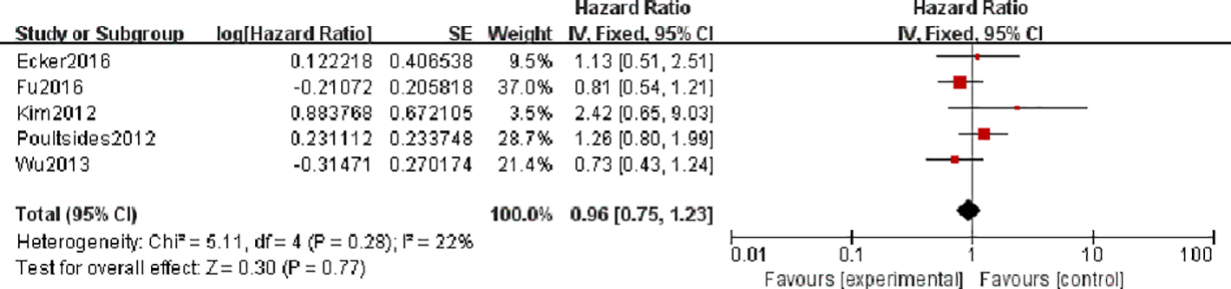
Forest plots to assess the effect of adjuvant therapy on the recurrence of patients with small bowel adenocarcinoma.

We hypothesized that RFS/DFS can be used as an indicator of relapse. Thus, the results of 7 relevant studies were combined to show that adjuvant therapy did not have a statistically significant effect on SBA recurrence after surgery (pooled HR = 0.89, 95% CI = 0.64–1.23, p = 0.48). The results did not change in the subgroup analysis of RFS and DFS (pooled HR = 1.19, 95% CI = 0.76–1.85, p = 0.44 and pooled HR = 0.62, 95% CI = 0.38–1.23, p = 0.06). No heterogeneity was observed in these three analyses (I^**2**^ = 11% and P = 0.48, I^**2**^ = 0% and P = 0.8, I^**2**^ = 0% and P = 0.47) (Fig 3).

### Subgroup analysis

We conducted a subgroup analysis for the results of pooled HRs of OS in patients with SBA according to country, scale of study, the method of adjuvant therapy, and statistical methods. The combined effect size showed that adjuvant therapy had a statistically significant effect on the OS of patients with SBA in Europe (pooled HR = 0.63, 95% CI = 0.5–0.8, p = 0.0002). However, no statistical significance was observed in Asia and America (pooled HR = 1.09, 95% CI = 0.61–1.94, p = 0.78 and pooled HR = 0.94, 95% CI = 0.74–1.2, p = 0.62, respectively). The result of the national database consolidation was pooled HR = 0.78, 95% CI = 0.58–1.04, p = 0.78 and pooled HR = 1.04, 95% CI = 0.84–1.29, p = 0.78 in the institution data subgroup. Nine univariate analysis studies showed that adjuvant therapy had a statistically significant effect on the IOS of patients with SBA (pooled HR = 0.81, 95% CI = 0.73–0.9, p = 0.0001). However, 5 multivariate analyses did not show similar results in this subgroup analysis (pooled HR = 0.81, 95% CI = 0.54–1.20, p = 0.29). Finally, the results of the AC and ACR subgroups were pooled HR = 0.84, 95% CI = 0.68–1.04, p = 0.11 and pooled HR = 1.19, 95% CI = 0.83–1.72, p = 0.35. Detailed pooled HRs and CIs of the subgroup analysis are displayed in Table 2.

**Table 2:** Results of pooled hazard ratios for overall survival according to subgroup analysis.

Subgroup		No. of patients	No. of studies	Combined results		Heterogeneity		Statistical Method	
				HR(95%CI)	p value	I^2^ (%)	P value		
**Overall survival**		5986	14	0.89[0.73,1.09]	0.25	62	0.001	Random model	
**Country**									
Asia		366	4	1.09 [0.61, 1.94]	0.78	60	0.06	Random model	
Europe		1439	5	0.63 [0.5, 0.8]	0.0002	19	0.29	Fixed model	
America		4241	5	0.94 [0.74, 1.20]	0.62	70	0.01	Random model	
**Analysis type**									
MU		3066	5	0.81 [0.54, 1.20]	0.29	78	0.001	Random model	
UV		2920	9	0.81 [0.73, 0.90]	0.0001	41	0.09	Fixed model	
**Treatment method**									
Chemotherapy		5722	10	0.84 [0.68, 1.04]	0.11	67	0.001	Random model	
Chemoradiation		264	4	1.18 [0.79, 1.77]	0.35	9	0.35	Fixed model	
**Data Sources**									
National		5135	3	0.78 [0.58, 1.04]	0.09	89	0.0001	Random model	
Institutional		851	11	1.04 [0.84, 1.29]	0.91	19	0.26	Fixed model	

MU: multivariate; UV: univariate; HR: Hazard Ratio.

### Relevant clinicopathological parameters

In order to further study the impact of clinical parameters on postoperative survival, we conducted a meta-analysis of six clinical parameters, which were age, sex, stage, differentiation, lymph node involvement, and margin status. The pooled HR shows that stage, differentiation, lymph node involvement and margin status were related to the OS of patients with SBA. However, age and sex had no effect on postoperative survival in patients with SBA. Detailed pooled HRs and CIs are displayed on Table 3.

**Table 3:** Results of pooled hazard ratios for overall survival according to clinicopathological parameters.

Outcome		No. of patients	No. of studies	Combined results		Heterogeneity		Statistical Method
				HR(95%CI)	p value	I^2^ (%)	P value	
**Age (>60 years)**		142	2	1.04 [0.30, 3.61]	0.95	74	0.05	Random model
**Gender**		3559	6	1.03 [0.94, 1.13]	0.51	34	0.18	Fixed model
**PD**		7157	6	2.19 [1.29, 3.70]	0.003	93	<0.00001	Random model
**positive margins**		4936	3	1.96 [1.71,2.24]	< .00001	0	0.54	Fixed model
**HTS**		317	3	1.72 [1.16, 2.57]	0.007	0	0.6	Fixed model
**NLNI**		130	2	0.09 [0.04, 0.22]	< .00001	35	0.21	Fixed model

PD: poorly differentiation; HTS: high tumor stage; NLNI: no lymph nodes involved; HR: Hazard Ratio.

### Sensitivity analysis

Sensitivity analysis was performed by the successive omission of individual studies to observe changes in heterogeneity. In this sensitivity analysis, we did not observe a great change in heterogeneity, which proved that no single study had significant heterogeneity and indicating that our analysis results are robust. Thus, heterogeneity in this study does not come from a single article.

### Publication bias

**Fig 4:**
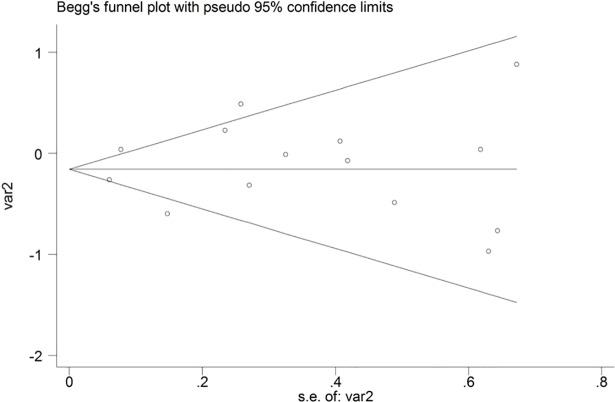
Begg`’s funnel plots to evaluate publication bias in related studies.

We performed publication bias analysis in 14 studies because 2 studies (Ecker2016 and Ecker2015) contained the same data analysis. Begg’s funnel plots did not reveal obvious asymmetry and the results were Begg’s test P = 0.584; Egger’s test P = 0.693. Therefore, there was no obvious publication bias in this meta-analysis (Fig 4).

## Discussion

Although SBA is a very rare malignancy, the incidence is gradually increasing. DA is the most common type of SBA [32]. Because of the increasingly poor prognosis, more postoperative treatment is required in patients with SBA. The most commonly used treatments are adjuvant chemotherapy and adjuvant chemoradiation in postoperative patients. Adjuvant therapy for SBA is a controversial issue, which requires further investigation by a general surgeon. The result of our meta-analysis has demonstrated that no associated survival benefit was conferred by the use of adjuvant therapy in patients with SBA. However, individual studies have also shown that adjuvant therapy has a significant survival benefit in patients with advanced or metastatic SBA [33, 34]. Unfortunately, because of the lack of high-quality research, especially randomized controlled trials (RCT), more research is needed to prove this conclusion.

In our review of the included studies, we found postoperative adjuvant chemotherapy may be better than adjuvant chemoradiation in SBA. Subgroup analysis of chemotherapy and chemoradiation also confirmed this idea (pooled HR = 0.84, 95% CI = 0.68–1.04, p = 0.11 vs. pooled HR = 1.19, 95% CI = 0.83–1.72, p = 0.35). {Postoperative chemotherapy may be more useful than chemoradiation. However, only 4 articles involving 264 patients on adjuvant chemoradiation were included in this subgroup study, so we need to perform more postoperative chemoradiation studies to confirm this conclusion. We found an interesting phenomenon that adjuvant therapy has a survival benefit for SBA patients in Europe, which may be because the included articles did not include adjuvant chemoradiation research. Although the American subgroup is not statistically significant, it still has the same trend as in Europe and the overall medical level in Asia may affect the outcome of Asian subgroups. In addition, we believe that multivariate analysis can eliminate the impact of related clinical factors and the results of multivariate analysis may be more credible. Thus, subgroup analysis and multivariate analysis may provide more accurate conclusions. In the relevant clinical parameters, poorly differentiated tumors, positive margins, and high tumor stage have a significant adverse effect on survival (pooled HR = 2.19, 95% CI = 1.29–3.7, p = 0.003, pooled HR = 1.96, 95% CI = 1.71–2.24, p<0.00001, pooled HR = 1.72, 95% CI = 1.16–2.57, p = 0.007), and no lymph node involvement has an associated survival benefit in patients with SBA (pooled HR = 0.09, 95% CI = 0.04–0.22, p<0.00001). We believe that these clinical parameters can be used as predictors of OS in patients with SBA. In addition, these clinical parameters can also be used as indicator of the effect of adjuvant therapy.

Related research shows that most adjuvant therapies are based on fluorouracil and FOLFOX (5-FU and oxaliplatin) is the most commonly used treatment, which is based on the treatment of other colorectal cancers. In addition, individual studies show no significant difference between the treatment regimens [18, 25]. However, related research does not provide enough evidence, so more studies should be performed to find the best treatment programs. The side effects of chemotherapy or radiotherapy should also be noted. Most of the side effects occur in the blood system and the most common hematological toxicity was neutropenia. Other toxicities include neurotoxicity, nephrotoxicity, and allergic reactions [25, 30]. Thus, we believe that postoperative adjuvant therapy should be used with caution when combined with the results of this analysis. Adjuvant radiotherapy is rarely used and related research is also rare. Therefore, no relevant analysis has been carried out in this article. The most effective chemotherapeutic regimen is still being explored and the commonly used chemotherapy regimen did not significantly improve postoperative survival [30]. In addition, studies have shown that the FOLFIRI regimen is an effective treatment for patients with advanced small bowel adenocarcinoma who are refractory to platinum-based chemotherapy [35]. So, need to explore more effective chemotherapy.

There are still some limitations in this meta-analysis. First of all, the quality of the included studies limited our research because they were all retrospective analyses. In general, the results of randomized controlled trials are more credible and the results of a meta-analysis consisting of RCTs are the most reliable. Second, subgroup analyses did not completely eliminate the effects of heterogeneity and sensitivity analysis also did not explain the source of heterogeneity. This shows that not all heterogeneity sources were considered in this meta-analysis. Although the random effects model was used to eliminate the heterogeneity, it also affected the results. In addition, we did not evaluate the role of adjuvant radiotherapy and performed limited research on adjuvant chemoradiation. Finally, we obtained the effect size according to the survival curves in 4 studies, which may have an impact on the results of our analysis.

## Conclusions

In this meta-analysis, we discovered that adjuvant treatment after surgery does not improve OS compared with only surgery in patients with SBA. In addition, poorly differentiated disease, positive margins, high tumor stage, and lymph node involvement had a marginally significant effect on survival in patients with SBA. Exploration of new adjuvant therapies and postoperative management is necessary.

## Supporting information

S1 Checklist PRISMA checklist. (DOC)

## Abbreviations

SBA: small bowel adenocarcinoma
HR: Hazard ratio
OS: overall survival
DA: duodenal adenocarcinoma
DFS: disease-free survival
RFS: relapse-free survival
5-FU: 5-fluorouracil
AT: adjuvant therapy
ACR: adjuvant chemoradiation
AC: adjuvant chemotherapy
MU: multivariate
UV: univariate
RCT: randomized controlled trials
