# Correction: Meta-analysis of postoperative adjuvant therapy for small bowel adenocarcinoma

**DOI:** 10.1371/journal.pone.0207816

**Published:** 2018-11-15

**Authors:** Xiaojian Ye, Guoqiang Zhang, Haibin Chen, Yong Li

One of the affiliations for the first author is not indicated. Xiaojian Ye is affiliated with: Department of Surgery, Shangrao First People's Hospital, Shangrao Jiangxi Province, China, and Department of General Surgery, The First Affiliated Hospital of Nanchang University, Yongwai Zhengjie, Nanchang, Jiangxi Province, China.
