# Correction: Correction: Meta-analysis of postoperative adjuvant therapy for small bowel adenocarcinoma

**DOI:** 10.1371/journal.pone.0209868

**Published:** 2018-12-20

**Authors:** Xiaojian Ye, Guoqiang Zhang, Haibin Chen, Yong Li

The affiliation for the first author is incorrect. Xiaojian Ye is not affiliated with #1 but with #2 Department of General Surgery, The First Affiliated Hospital of Nanchang University, Yongwai Zhengjie, Nanchang, Jiangxi Province, China.
